# Global Drivers of Phytoplankton Phenology Trends

**DOI:** 10.1111/gcb.70955

**Published:** 2026-06-10

**Authors:** Joe K. Guest, Marie‐Fanny Racault, Corinne Le Quéré

**Affiliations:** ^1^ School of Environmental Sciences University of East Anglia Norwich UK

## Abstract

Phytoplankton are key contributors to global primary production and serve as sensitive indicators of changes in marine ecosystems. Changes in phytoplankton phenology, and in particular the onset, termination, and duration of their growing period, have important implications for higher trophic levels that feed on them. Several local studies have provided evidence linking phenological shifts to climate change. However, global analyses have so far relied on time‐series observations of surface chlorophyll covering less than two decades, making it difficult to separate trends from variability and establish the coherence of underlying drivers at the global level. Here we use the OC‐CCIv5 satellite‐derived observations harmonised across satellite sensors and the NEMO‐PlankTOM12 global ocean biogeochemical model to revisit recent trends in phytoplankton phenology and identify their global drivers during the 1998–2020 period. Using statistical analysis to separate and control for covariance among underlying drivers, we show that SST warming trends are clearly associated with earlier initiation, later termination, and longer duration of the phytoplankton growing season. In contrast, changes in MLD, SST variability, and chlorophyll are associated with shifts in initiation and termination in the same direction, such that both boundaries move in parallel, partly cancelling their effect on duration. The PlankTOM12 model successfully reproduces most of the observed patterns in phenology and their association with the underlying drivers, providing evidence of the mechanistic relationships, and helping to ascertain the global significance of the drivers. Our findings suggest that trends towards earlier initiation, later termination, and longer duration of phytoplankton growing periods are likely associated with climate change, and may therefore persist in the near future under continued global warming.

## Graphical abstract

Observations and a global biogeochemical model were used to examine recent shifts in phytoplankton phenology at the global scale. Sea surface temperature trends emerge as the strongest predictor of these changes, relative to the other drivers considered, and are associated with earlier initiation, later termination, and longer growing periods.

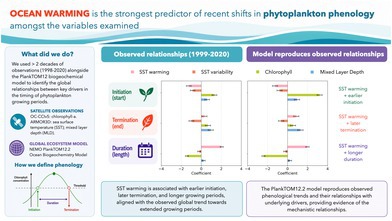

## 1 Introduction

Phytoplankton contribute almost half of global net primary production (NPP) (Behrenfeld 2014; Kulk et al. 2020), fundamentally supporting marine ecosystems. Their seasonal biomass differs regionally in timing and amplitude, driven by distinct environmental conditions (Racault et al. 2012). At high latitudes, phytoplankton experience short, intense growing periods forming prominent spring blooms, initiated either by increased light availability following the shallowing of the mixed‐layer during the transition from winter to spring (Sverdrup 1953; Chiswell 2011; Taylor and Ferrari 2011) or reduced grazing due to predator dilution when the mixed‐layer is at its deepest in late‐winter (Behrenfeld 2010; Behrenfeld et al. 2013; Sallée et al. 2015). Bloom termination at high latitudes occurs predominantly through increased grazing and nutrient limitation (Ryan‐Keogh et al. 2023; Thomalla et al. 2023). Conversely, phytoplankton initiation and termination at low and mid latitudes follows more directly the seasonal cycle of stratification and associated nutrient availability (Friedland et al. 2018; Gittings et al. 2018; Racault, Sathyendranath, Brewin, et al. 2017; Racault et al. 2015).

Phytoplankton growing periods strongly influence marine food webs, such that regional shifts in bloom timing can result in trophic mismatches, eventually causing species extirpation (Asch et al. 2019; Cushing 1959; Edwards and Richardson 2004; Platt et al. 2003; Vikebø et al. 2021). Mismatches between primary and tertiary production have already been documented, negatively affecting copepods in the Beaufort Sea (Dezutter et al. 2019) and fish recruitment in the Red Sea (Gittings et al. 2021). As ocean warming intensifies, trophic mismatches are expected to become more common. One model study suggested high‐latitude fish recruitment failures could increase tenfold under a very high emissions scenario (RCP 8.5; Asch et al. 2019). In tropical reef ecosystems, phytoplankton form a crucial food source for sponges, bivalves and pelagic larvae (Gittings et al. 2018). Therefore, changes in phytoplankton phenology combined with observed poleward range shifts (Beaugrand et al. 2015; Cooley et al. 2022) may also have severe consequences for low‐latitude ecosystems. Additionally, poleward shifts in species distributions may lead to ecosystem restructuring, resulting in altered phenology as high‐intensity short growing period regions shift to prolonged, lower‐intensity growing patterns characteristic of mid‐latitude ecosystems (Chivers et al. 2020; Gregg et al. 2017; Cooley et al. 2022).

Although many studies have successfully linked regional shifts in phytoplankton phenology to local environmental factors including sea surface temperature (SST), nutrient supply, grazing pressure, and mixed‐layer depth (MLD), their dominant modes often vary considerably between ocean basins and latitudes (Friedland et al. 2018; Gittings et al. 2018; Ardyna and Arrigo 2020; Racault et al. 2012). For instance, elevated SSTs have been reported to lead to earlier bloom initiation and prolonged phytoplankton growing periods in temperate and high‐latitude regions such as the North Sea and subarctic North Pacific (Chivers et al. 2020; Yamaguchi et al. 2022; Ardyna and Arrigo 2020). In contrast, elevated SSTs have been linked to weaker and shorter winter blooms in tropical regions such as the Red Sea (Gittings et al. 2018). Furthermore, previous studies examining global relationships between SST and phenology have yielded conflicting results, with some finding minimal or negative associations between SST and bloom duration (Friedland et al. 2018), highlighting the complexity of isolating primary global‐scale drivers from multiple interacting environmental variables.

A critical limitation to detect global phytoplankton phenological trends is the length of observational datasets. Previous research using CMIP5 models suggested that for mean phytoplankton biomass or primary production, approximately three decades of observations may be required to distinguish climate‐driven trends from interannual variability (Henson et al. 2016). Consequently, short datasets have impeded the conclusive identification of global‐scale drivers, resulting in many analyses limited to specific climate events such as ENSO cycles (Racault, Sathyendranath, Menon, and Platt 2017). However, more recent work demonstrates that climate trends can emerge from just two decades of satellite‐derived ocean colour data (Cael et al. 2023), owing to the multivariate nature of remote‐sensing reflectance (Rrs) and the comparatively low interannual variability in certain wavebands. In this study, we overcome these limitations by utilising the longest observational satellite record spanning 23 years (1998–2020). Importantly, we explicitly separate the effects of SST trends from interannual SST variability and other drivers, rather than solely examining linear trends in phytoplankton phenology. This approach allows for a robust estimation of how warming trends are independently associated with phenological change at a global scale. By adopting this method, we aim to provide clearer insight into how climate‐driven shifts in SST and associated environmental factors shape phytoplankton phenology. We further use the PlankTOM12 global biogeochemical model to provide additional insights into how the phytoplankton phenology may respond to both physical and biogeochemical changes, and demonstrate the coherence of the findings.

In this paper, we use two decades of satellite and model data to provide an updated assessment of observed trends in phytoplankton phenology and identify the key environmental drivers responsible for those trends at the global level. After validating the global spatial patterns and temporal dynamics of phytoplankton growing periods in the PlankTOM12 model data against satellite‐derived observations, we use statistical analysis to isolate the primary drivers. Our analysis combines observational and modelled datasets. Satellite observations constrain recent changes in phytoplankton phenology, whereas the PlankTOM12 ecosystem model provides a mechanistic framework to examine whether the relationships between environmental drivers and phenology inferred from observations emerge from simulated ecosystem dynamics, and to assess whether similar phenological trends arise in simulations with time‐varying versus constant climate forcing.

## 2 Methods

### 2.1 Observational Data

The European Space Agency (ESA) Ocean Colour‐Climate Change Initiative version 5 (OC‐CCIv5; Sathyendranath et al. 2019) daily (1998–2020) chlorophyll‐a data and associated bias calculations with a global area coverage at 4‐km resolution were downloaded from https://climate.esa.int/en/projects/ocean‐colour/. The OC‐CCIv5 dataset merges data from five satellites (SeaWiFS, MODIS, VIIRS, OLCI and MERIS) and is a validated and error‐characterised climate‐quality‐controlled chlorophyll‐a concentration product.

The 23‐year dataset was preprocessed through the following steps. A bias correction was first applied using the gridded bias estimates provided with the OC‐CCIv5 product, following the methodology outlined in the user manual, to remove known sensor and processing biases. The data were then spatially binned from the native 4 km resolution to a 1° × 1° regular latitude–longitude grid by averaging all observations within each bin. Missing values were filled using Ferret's ‘FILL_XY’ function, which performs iterative low‐order 2D smoothing by averaging over neighbouring valid values; five iterations were applied, as determined through sensitivity testing against model data to minimise the risk of bias introduction. A 21‐day temporal box‐smoothing filter was subsequently applied, following the approach of Racault et al. (2012), to reduce short‐term variability and enhance the detection of phenological signals. Finally, data poleward of 65° N and 65° S were excluded due to persistent cloud cover, sea ice, and seasonal low light conditions, which limit the reliability of satellite ocean colour retrievals in these regions (Ferreira et al. 2022). An additional 2° buffer was removed along coastlines, consistent with OC‐CCIv5 recommendations, as these areas exhibit the highest retrieval bias and are less representative of the open ocean.

To identify spatial patterns of phytoplankton phenology, we created a 23‐year climatology using data from 1 January 1998 to 31 December 2020. To examine trends in phenology metrics over time, we constructed two time series of biennial (2‐year) climatologies, each point representing the average seasonal cycle calculated from two consecutive years of data. These produced two offset time series of 11 data points: (i) 1998–1999, 2000–2001, …, 2018–2019 and (ii) 1999–2000, 2001–2002, …, 2019–2020. This approach avoids imposing an arbitrary calendar‐year boundary while also allowing trends to be evaluated using two independent offset series.

Monthly SST and MLD variables from the Multi Observation Global Ocean ARMOR3D L4 analysis were downloaded from E.U. Copernicus Marine Service Information (https://doi.org/10.48670/moi‐00052; Guinehut et al. 2012; Mulet et al. 2012). Variables were regridded from a ¼° to 1° resolution. For SST an average of the top 3 depth boxes were taken, representing the surface 0–10 m, and corresponding to the top model level. The MLD used in this analysis represents the depth at which the temperature decreases by 0.2°C compared to the surface. Using the same methodology as applied to the chlorophyll‐a data, biennial climatologies were constructed for SST and MLD.

### 2.2 The NEMO‐PlankTOM12 Model

The PlankTOM12 model is used here to help with the interpretation of observed satellite‐derived trends in phenology. PlankTOM12 is a global ocean biogeochemistry model that represents ecosystem dynamics by grouping organisms into 12 plankton functional types (PFTs), chosen for their contribution to the carbon cycle (Le Quéré et al. 2005). PFTs include six phytoplankton (picophytoplankton, N_2_‐fixers, coccolithophores, mixed‐phytoplankton, diatoms, *Phaeocystis*), bacteria/Archaea (herein called simply bacteria), and five zooplankton (protozooplankton, pteropods, mesozooplankton, crustacean macrozooplankton and gelatinous zooplankton). PlankTOM12 integrates the 10 PFTs as described in Le Quéré et al. (2016), with the pteropod PFT as described in Buitenhuis et al. (2019), and the gelatinous zooplankton PFT described in Wright et al. (2021). Compared to its last published version in Friedlingstein et al. (2025), the latest PlankTOM12 model (PlankTOM12.2) includes a sediment pool, updates to organic carbon, inorganic carbon and silica remineralisation, a 3‐parameter formulation for PFT growing rates (Guest 2023).

PlankTOM12 represents 39 biogeochemical tracers including complete marine cycles of carbon, phosphorus, silicon, alkalinity and oxygen, and simplified cycles of nitrogen and iron. PFT growth and loss processes include primary production (for phytoplankton), respiration, grazing and faecal production (for zooplankton), and mortality. Sedimentation processes are included by distinctly representing two types of particulate organic carbon (POC) based on size. Small POC sinks at a constant rate of 3 m per day, whereas the larger POC sink at variable speed, from 3 to 150 m per day, modulated by the ballasting effect of their mineral composition (Buitenhuis et al. 2013). The two POC are available as food source for zooplankton, which result in both aggregation and disaggregation. Additionally, the model incorporates the representation of a dissolved organic carbon (DOC) component. Bacteria remineralise all organic components explicitly. Model parameters are based on observations where available (e.g., Buitenhuis et al. 2006, 2010, 2019; Le Quéré et al. 2016; Wright et al. 2021), with loss terms and feeding preferences also guided by the relative predator/prey ratio and the model fit to global PFT carbon biomass based on observations (Maredat; Buitenhuis et al. 2013) and surface chlorophyll (OC‐CCIv5; Sathyendranath et al. 2019). River fluxes of nutrients and organic and inorganic carbon are injected into the ocean at river mouths and are correspondingly removed evenly from the sediment layer to conserve mass balance. A full description of the model is provided in the PlankTOM12 manual including parameter units and values (http://doi.org/10.5281/zenodo.8388158).

PlankTOM12 is embedded in the global ocean general circulation model Nucleus for European Modelling of the Ocean version 3.5 (NEMO v3.5), with the LIMv2 thermodynamic‐dynamic sea‐ice model (Madec 2012; Iovino et al. 2013). The NEMO model is projected onto a tripolar orthogonal curvilinear ocean mesh with a horizontal resolution of 2° longitude and 1.5° latitude on average, enhanced in the tropics and at both poles. The model represents 31 vertical levels, which decrease in resolution from 10 m between 0 and 100 m to 500 m at depth. Model simulations are initialised with observations from the World Ocean Atlas (Levitus et al. 2009) for NO_3_, PO_4_, SiO_3_, O_2_, temperature and salinity (Garcia et al. 2018b, 2018a; Locarnini et al. 2018; Zweng et al. 2018). Dissolved inorganic carbon (DIC) and alkalinity are from GLODAPv2 (Olsen et al. 2016), with DIC adjusted to the start year (1750) by removing its estimated anthropogenic component (Keppler et al. 2020).

Simulations are forced with daily wind stress, precipitation, cloud cover, and surface air temperature from NCEP reanalysis data (Kalnay et al. 1996). Temperature is restored using monthly mean fields from NOAA OISST v2.1 from 1982 onward and from WOA18 monthly climatologies prior to 1982 (Locarnini et al. 2018), whereas salinity restoring uses decadal monthly climatologies from WOA18 (Zweng et al. 2018). We conduct two parallel simulations: a simulation with variable climate conditions corresponding to each year from 1948 to 2020, and a control simulation with climate conditions looping over the same single year (1990). This allows us to verify that phenological changes do not emerge in the absence of time‐varying climate forcing, confirming that they are not driven by model drift or spurious internal variability. Both simulations are initialised in 1750, with a spin‐up period from 1750 to 1947. This ensures that are no drifts resulting from the simulation set up.

SST, MLD, and surface chlorophyll concentration were obtained from the PlankTOM12 model and sampled at the latitude, longitude, and time points corresponding to the satellite observations.

### 2.3 Phenological Indicators

Phenology indices are metrics calculated from surface chlorophyll concentrations to identify the timing of the growing periods of phytoplankton in the ocean. Phenology indices are used because they are relative metrics that are influenced less by the differences in satellite sensors across the time series and are therefore a more accurate representation of ecosystem shifts (Platt and Sathyendranath 2008). The phenological indices used in this paper are taken from Racault et al. (2012). First, the timing of the peak chlorophyll ($b_{m}$), defined as the timing of the maximum chlorophyll value in the seasonal cycle, is identified. The maximum chlorophyll concentration ($b_{a}$) is then defined as the highest chlorophyll concentration in a biennial climatology. To determine the timing of initiation ($b_{i}$) and termination ($b_{t}$), we searched before and after $b_{m}$, for the points at which chlorophyll crossed the threshold. The duration of the growing period ($b_{d}$) is defined as the number of days between the initiation and termination dates. The threshold was defined as the median chlorophyll concentration at each grid cell plus 5%, to be consistent with previous studies (e.g., Siegel et al. 2002; Racault et al. 2012). Other studies, such as Friedland et al. (2018), use a sequential averaging algorithm (STARS), but as fewer studies implement this method, it is less comparable with previous work. The choice of 5% for the median threshold is unlikely to impact results, as Siegel et al. (2002) showed there was little difference between phenology metrics calculated using thresholds ranging between 1% and 30%. However, unlike previous studies, the median of each biennial climatology is used here rather than the long‐term median chlorophyll concentration to prevent increases in the chlorophyll concentration resulting in increases in growing period duration ($b_{d}$). The threshold method only highlights the characteristics of the dominant phytoplankton growing period, which correspond to the growing period associated with the maximum chlorophyll concentration in the annual cycle. In‐depth reviews comparing the methodology used to estimate phenological indices are available in Brody et al. (2013) and Nicholson et al. (2025).

In the present study, phenology indices were calculated using daily average data from OC‐CCI and from the PlankTOM12 model. Data within two degrees of the coast and beyond 65° N or 65° S were excluded due to biases and incomplete observational coverage in coastal and polar regions. Furthermore, to prevent detection of large trends during a change in Julian date across the calendar boundary, that is, between January (Day 1) and December (Day 365), the dates of initiation, peak chlorophyll, and termination were standardised. For each pixel, dates with growing periods were first identified across all years of the time series. The non‐growing period was defined as the longest continuous period without growing activity in any year, and phenological dates for that pixel were then temporally rotated relative to the midpoint of this period.

### 2.4 Statistical Methods

The trends were calculated over the full time period for each pixel using Theil‐Sens slopes. Sens‐slope trends are used as a robust alternative to linear regression because the data are susceptible to outlier bias due to interannual variability (Salgado‐Hernanz et al. 2019). The coherence of spatial patterns in trends is discussed regardless of the significance of individual grid‐cells.

The individual contributions of SST trend, SST variability (defined as the linearly detrended SST time series), MLD, and surface chlorophyll concentration to trends in phenological indices were assessed via grid‐cell level multiple regression, with small‐sample robust standard error adjustments applied following Long and Ervin (2000). Unlike phenology metrics based on fixed chlorophyll thresholds, which can be directly influenced by changes in chlorophyll magnitude, the indices used here are defined relative to a moving biennial baseline. This reduces the direct dependence of the phenology metrics on absolute chlorophyll concentration and allows chlorophyll to be included as a predictor of phenological variability associated with the seasonal development of the dominant growing period. However, given the known interdependencies among these predictors, particularly the influence of SST on both MLD and chlorophyll, variance inflation factors (VIFs) were computed to evaluate collinearity. To minimise its influence on global coefficient estimates, we applied a masking procedure whereby cells with VIF greater than 5 were excluded prior to spatial aggregation. After masking, global slope estimates for each predictor reflect only regions of low multicollinearity. This filtering removed 16.2% and 23.1% of ocean grid cells across predictors in the observations and model datasets, respectively.

Global average estimates (${\hat{\beta }}_{\text{global}}$) were derived using a weighted median approach, with weights determined by the cosine of latitude ($\cos\left(\varphi_{i,j}\right)$) to account for varying grid cell areas. The variance for the global slopes was approximated by aggregating local variances, weighted by the squared latitude‐based weights, providing the global standard error ($\sigma \left({\hat{\beta }}_{\text{global}}\right)$).

Global averages for phenological trends and their relationships with environmental drivers were determined by first calculating the slopes $\beta_{i,j}$ and their corresponding standard errors $\sigma_{i,j}$ for each valid grid cell (i,j). The statistical significance of the global value is evaluated using its standard error to compute a *z*‐score $\left(z=\frac{\hat{\beta }\text{global}}{\sigma \left(\hat{\beta }\text{global}\right)}\right)$. This score is used to calculate a two‐tailed *p*‐value, determining the likelihood of observing such a trend if there were no true underlying global effect ($p=2\times \left(1-\Phi \left(|z|\right)\right)$).

Finally, the 95% confidence interval was calculated for the global trend, using the standard critical value and the computed standard error, to provide a range that is likely to contain the true global trend (${\hat{\beta }}_{\text{global}}\pm z\;0.975\times \sigma \left({\hat{\beta }}_{\text{global}}\right)$, with z0.975≈1.96).

OpenAI's ChatGPT (GPT‐4o/5) language model was used to assist in selected coding tasks and figure construction. All outputs were critically reviewed, edited, and validated by the authors.

## 3 Results

### 3.1 Spatial Patterns in Phenology

**FIGURE 1:**
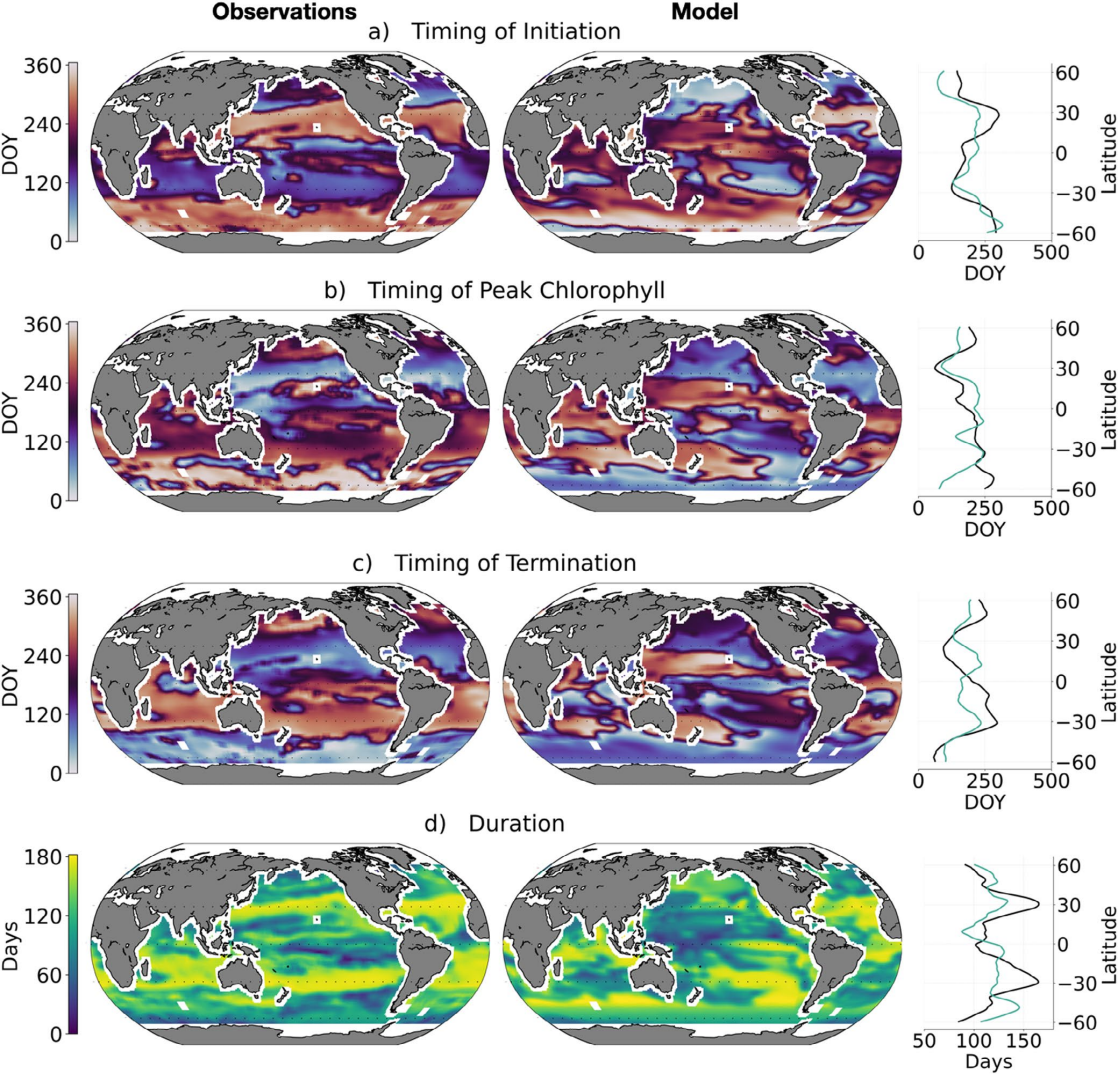
(a–d) Global climatological spatial patterns of phytoplankton phenology indices derived from the OC‐CCIv5 satellite observations (left) and the PlankTOM12.2 model (centre), averaged over 1998–2020. Panels show (a) timing of initiation in day of the year (DOY), (b) timing of peak chlorophyll (DOY), (c) timing of termination (DOY) and (d) duration of the growing period in days. For timing variables (initiation, maximum timing, and termination), colours represent day of year using a cyclical colour scale, such that values near the start and end of the calendar year are represented by the same colour. The right hand panels show the zonal average of each phenological index from OC‐CCIv5 (black) and PlankTOM12.2 (green) smoothed with a 5° running mean. Values exceeding 365 DOY in the zonal means represent growing periods that cross the calendar year boundary, for example, 400 ≈ February and 500 ≈ April.

Here, we compare the global phenological patterns of phytoplankton phenology calculated from the OC‐CCIv5 satellite data and the PlankTOM12 model. Globally, phytoplankton phenology shows longer growing periods starting later in the year in the subtropics, compared to the subpolar regions (Figure 1). The model broadly reproduces the latitudinal averages of growing period phytoplankton phenology, but with some regional differences (Figure 1).

In satellite‐derived observations, growing periods occur between October and March in the Northern Hemisphere around 30° N, and between May and October in the Southern Hemisphere around 30° S (Figure 1). The shift between the timing of the Northern and Southern Hemisphere phenological patterns is relatively smooth, although there is regional patchiness in the transition around the equator. Although observations indicate that growing periods in the South Pacific typically begin around April–May, the model displays considerable regional variability, with initiation dates ranging from March to October.

The model is similar to the observations regarding the transition of initiation, timing of peak chlorophyll, and termination of growing periods through these latitudes. Despite some discrepancies, the model demonstrates a similar gradient between the subpolar‐ and subtropical‐latitudes from spring to winter initiation dates as shown in observations. This suggests that the model reproduces shifts in the drivers influencing growing limitation, from light limitation and grazer density in the poles to nutrient limitation in the tropical‐ and subtropical‐latitudes with some accuracy. Termination timing is typically difficult to reproduce in global ecosystem models because it depends on the balance between phytoplankton growth and multiple loss processes (grazing, remineralisation and nutrient depletion), which are strongly influenced by ecosystem structure and grazer parameterisation (Karakuş et al. 2022). In contrast, the timing of growing period initiation and peak chlorophyll is more directly linked to phytoplankton growth responses to abiotic forcing, which are usually better constrained by observations (Le Quéré et al. 2016).

The global mean growing period duration in satellite‐derived observations is approximately 122 days (Figure 1d). Regionally, the shortest durations occur in the subpolar zones, with an average of 92 days in the north and 105 days in the south. In the tropics, durations are slightly longer, averaging 112 days. The subtropics exhibit the longest growing periods, with mean durations of 142 days in the north and 138 days in the south. PlankTOM12.2 produces a global mean growing period duration of 120 days, broadly consistent with observations. In addition, the model has similar zonal patterns: the longest growing period duration occurs in the subtropical regions and the shortest growing period duration occurs in the tropical pacific. However, there are notable regional differences. In the subpolar regions, the model overestimates duration, simulating 120 days in the north and 116 days in the south, longer than the observed 92 and 105 days, respectively. Overall, the model aligns well with observed patterns in growing period duration, despite regional biases.

**FIGURE 2:**
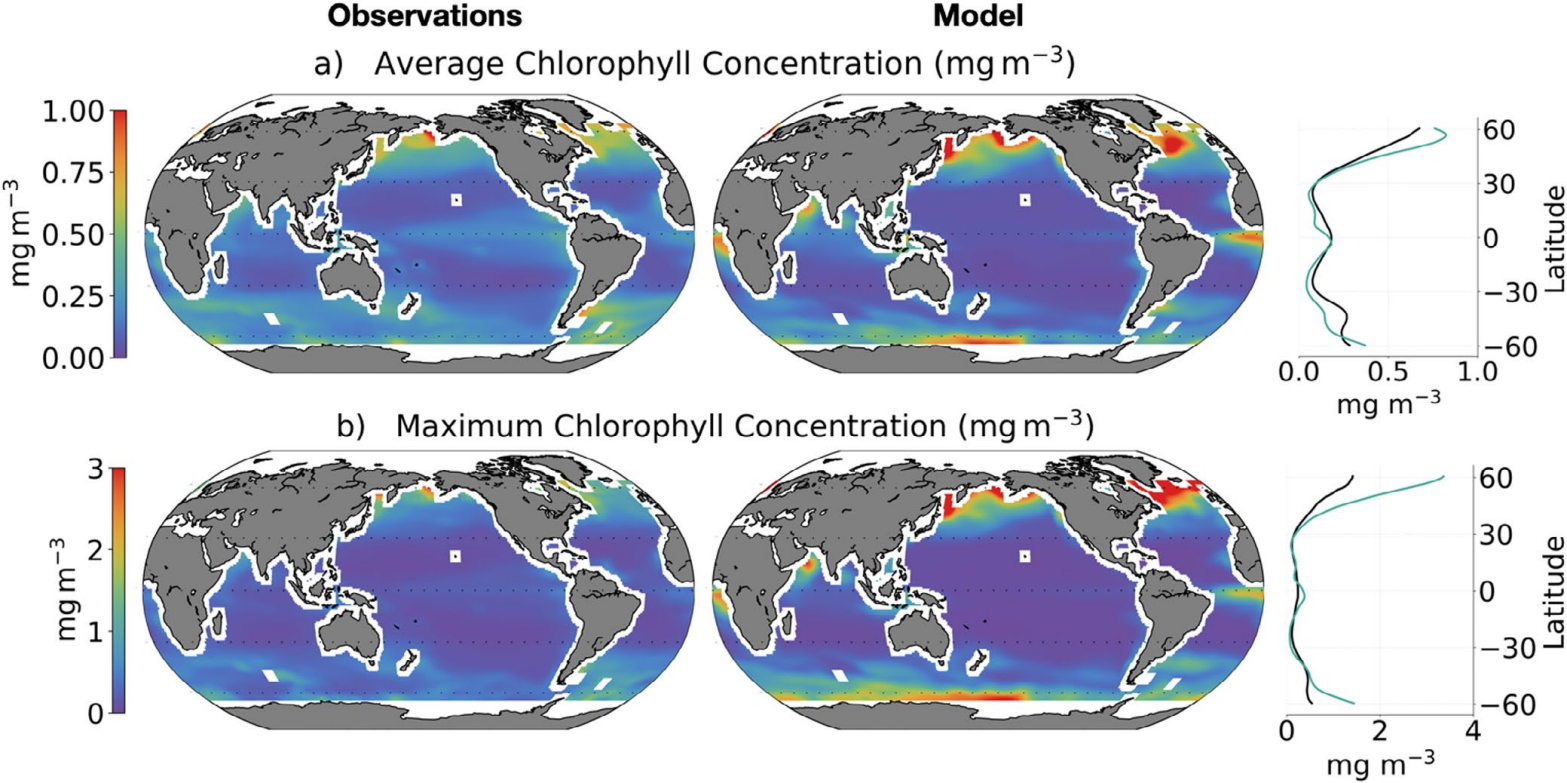
(a, b) Global climatological spatial patterns of (a) mean chlorophyll concentration (mg m^−3^) and (b) maximum chlorophyll concentration (mg m^−3^), averaged over 1998–2020. Each row shows the OC‐CCIv5 satellite observations (left) and the PlankTOM12.2 model (centre), and their zonal means (right; OC‐CCIv5 in black and PlankTOM12.2 in green) smoothed with a 5° running mean.

Satellite‐derived observations show clear latitudinal patterns in both average chlorophyll concentration and maximum chlorophyll concentration. Latitudinally averaged chlorophyll is highest in the subpolar regions (0.75 mg m^−3^ in the north and 0.37 mg m^−3^ in the south), intermediate in the tropics (0.21 mg m^−3^), and lowest in the subtropical gyres (0.07 mg m^−3^ in both hemispheres; Figure 2a). Maximum chlorophyll concentration follows a similar meridional structure, with observed latitudinal averages of 1.51 mg m^−3^ in the subpolar north, 0.79 mg m^−3^ in the subpolar south, and much lower amplitudes in the subtropics (0.11 mg m^−3^) and tropics (0.29 mg m^−3^). In low‐productivity oligotrophic gyres such as the subtropical Pacific, chlorophyll levels are both low and seasonally stable, with relatively uniform spatial patterns in both average and maximum chlorophyll concentration.

PlankTOM12.2 broadly captures these large‐scale latitudinal patterns, reproducing elevated chlorophyll concentrations in the subpolar regions and reduced values in the subtropics and tropics (Figure 2a,b). Although the model follows the broad spatial structure in oligotrophic gyres, it overestimates seasonal variability in the high latitudes. This is especially apparent in the subpolar North where the model simulates comparable or slightly elevated mean concentrations (0.5–0.85 mg m^−3^), but substantially higher maximum chlorophyll values, frequently exceeding 2–3.5 mg m^−3^. As a result, the difference between mean and maximum chlorophyll concentration is markedly larger in the model than in the observations at high northern latitudes, indicating an enhanced seasonal amplitude in the simulated chlorophyll field. Despite these regional discrepancies, PlankTOM12.2 demonstrates good agreement with satellite observations at the level of latitudinal averages, successfully reproducing the large‐scale meridional gradients in both average concentration and maximum chlorophyll concentration (Figure 2). In addition, differences in magnitude between the model and observations do not prevent the model from reproducing phenological patterns. This is because the phenological indices are defined using a moving chlorophyll threshold rather than absolute chlorophyll concentration. As a result, they capture the timing of bloom dynamics rather than the size of the seasonal changes, and are therefore less affected by biases in seasonal amplitude.

### 3.2 Global Mean Trends in Phenology

**FIGURE 3:**
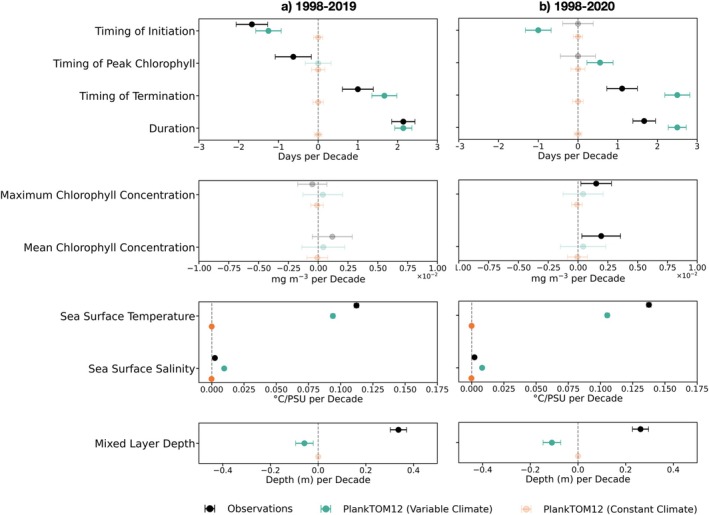
Global average trends per decade in phenology indices, chlorophyll metrics, and environmental drivers, derived from 11 biennial climatologies over 1998–2019 (left) and 1999–2020 (right). Phenology indices include timing of initiation, timing of the maximum, timing of termination (all in days decade^−1^), and duration (days decade^−1^). Chlorophyll metrics are maximum chlorophyll concentration and mean concentration (mg m^−3^ decade^−1^). Environmental drivers are sea surface temperature (°C decade^−1^), sea surface salinity (PSU decade^−1^), and mixed‐layer depth (m decade^−1^). Black symbols denote observational datasets: OC‐CCIv5 satellite‐derived observations for phenology and chlorophyll metrics, and ARMOR3D Copernicus Marine Service reanalysis products for the environmental drivers. Green symbols show PlankTOM12.2 model results with variable climate forcing and orange symbols show PlankTOM12.2 model results with constant climate forcing. Darker colours show significant trends (p<0.05).

Significant trends in global mean phenology are observed over the 1998–2020 period examined here (Figure 3). A trend towards earlier initiation by −1.67 ± 0.40 days per decade is observed, but only in one of the two biennial climatologies. A trend towards later termination of 1.00 ± 0.39 and 1.25 ± 0.38 days per decade, respectively, is observed in both climatologies. The opposing trends between earlier initiation and later termination drive increases in duration of 2.14 ± 0.29 and 1.67 ± 0.29 days per decade, which are significant in both observed climatologies (Figure 3).

The PlankTOM12.2 model reproduces the direction of the global mean trends for most of the indices for initiation, termination, and duration (Figure 3), in model simulations forced with variable climate conditions. The model shows trends towards earlier initiation by −1.25 ± 0.32 and −1.00 ± 0.32 days per decade, respectively for each biennial climatology. It also produces opposing trends in termination (1.67 ± 0.31 and 2.50 ± 0.31 days per decade), which drive increases in duration of 2.14 ± 0.21 and 2.50 ± 0.23 days per decade (Figure 3). The model simulation forced with constant climate conditions shows no significant trends in phenology indices, as expected (Figure 3).

The presence of significant trends in termination in both biennial climatologies, across both observations and model simulations, suggests that termination timing plays a substantial role in driving changes in growing period duration. As termination is sometimes omitted from phenological analyses (Friedland et al. 2018; Henson et al. 2017), these results highlight the importance of including it as a core metric when assessing changes in the growing period.

Trends in the maximum chlorophyll concentration of the bloom ($b_{a}$) are less conclusive. Results show only one of the observed biennial climatologies shows a significant global trend 0.0015 ± 0.0013 $\mathrm{mg}\;m^{-3}$, with no significant trend present in the model data. There are no significant trends in the mean chlorophyll concentration present in the satellite derived chlorophyll or model data.

Both delineated datasets are used here to show that there is general agreement between the two time series. However, for brevity, only results from the time series between 1999 and 2020 are shown throughout the remainder of this paper. This time series is chosen because there are significant gaps in the first year of data, with only 10.5e6 points of good data in 1998 compared to an average of 12.5e6 points of good data in the whole time series. In addition, these trends describe changes in phenological indices over the selected time periods and do not explicitly account for concurrent changes in environmental drivers such as temperature or mixed‐layer depth. Differences between periods may therefore reflect the influence of interannual variability, including strong climate events such as the 1997–1998 El Niño, acting alongside a longer‐term trend. The relative contributions of temperature trends and variability are evaluated separately using the regression framework described in Section 3.4.

### 3.3 Spatial Trends in Phytoplankton Phenology

**FIGURE 4:**
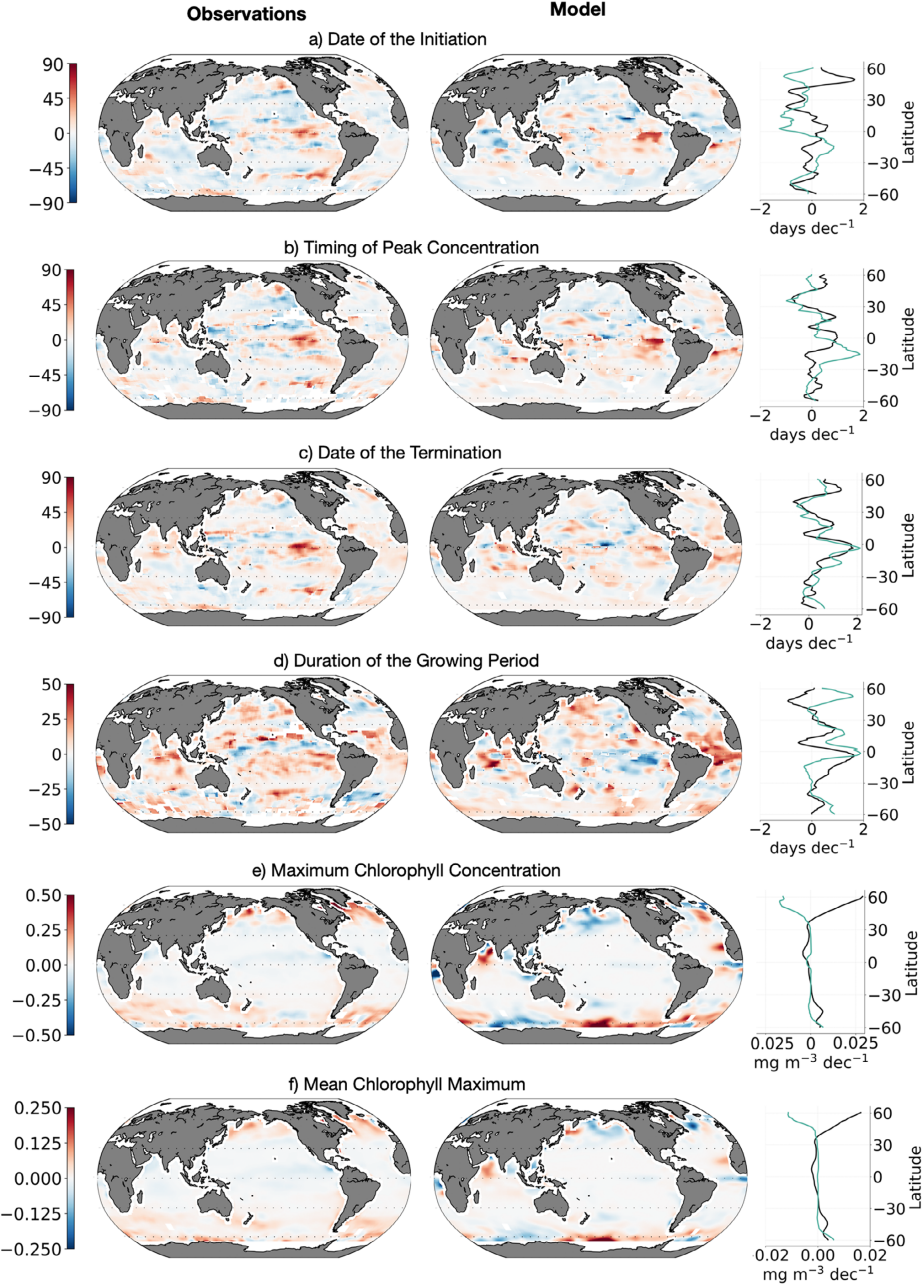
(a–f) Theil‐Sen trends in phytoplankton phenology indices and chlorophyll metrics calculated from 11 biennial climatologies (1999–2020). Spatial trend patterns are shown for OC‐CCIv5 satellite observations (left) and the PlankTOM12.2 model (centre). The right‐hand panels show 10°‐smoothed zonal‐mean trends for OC‐CCIv5 (black) and PlankTOM12.2 (green). Panel shows (a) timing of initiation, (b) timing of peak chlorophyll, (c) timing of termination and (d) duration of the growing period (all in days decade^−1^) and (e) maximum chlorophyll concentration and (f) mean chlorophyll concentration (both in mg m^−3^ decade^−1^).

The grid‐level trends of the phenology indices calculated from observations and the PlankTOM12 model are examined to investigate the spatial patterns underlying their global mean trends. This analysis is not limited to assessing model–data differences, which are relatively small, but also highlights the strong coherence between the two and how these consistent spatial structures contribute to the global signal (Figure 4). The zonal averages of the trends are also provided to facilitate the comparison. Trends in the timing of initiation, peak concentration, termination and duration of the growing period in the model simulation generally follow those derived from observations in the 40° N–40° S latitudinal band, with greater differences poleward of 40°.

Satellite‐derived observations reveal global trends over the past two decades towards earlier initiation, earlier timing of peak chlorophyll, and later termination of the growing period (Figure 3). These trends are, however, spatially heterogeneous. Later initiation is concentrated in the subpolar North, the tropical Pacific, the western Indian Ocean, and parts of the Southern Ocean (Figure 4). As a result, while most regions exhibit a lengthening of the growing period, localised contractions occur in the North Atlantic, along the eastern boundaries of the Atlantic and Pacific off South America, and in parts of the tropical Pacific and western Indian Ocean (Figure 4).

PlankTOM12.2 broadly reproduces the spatio‐temporal patterns of phenological change observed between 1999 and 2020 including widespread increases in growing period duration. In the subpolar North, for example, observations show delayed timing of initiation, peak chlorophyll and termination, whereas the model simulates earlier initiation and delayed termination, resulting in a greater increase in duration (15 vs. 5 days per decade). Similarly, in the Southern Ocean, termination is later by 7.3 days per decade in the model compared to 2.3 days per decade in observations. The model also captures the general latitudinal structure of phenology trends. In both the model and observations, the strongest trends occur in the tropics and subpolar regions, with growing period duration increasing by up to 75 days per decade in observations and 68 days per decade in the model near the equator, representing phenological shifts of several months, rather than small adjustments in bloom timing. Despite regional mismatches in timing and magnitude, the model captures the dominant spatial and zonal features of observed phenological change. The interpretation of drivers presented later focuses on the relationships between phenology and environmental variables rather than the exact magnitude of regional trend estimates (Section 3.4).

Trends in chlorophyll concentration metrics vary considerably across regions, despite both maximum chlorophyll concentration and mean chlorophyll concentration exhibiting no significant global trends between 1998 and 2020 (Figure 4). In the Southern Ocean, both PlankTOM12.2 and the observations show predominantly positive trends in mean and maximum chlorophyll concentration. However, in the subpolar North, trends in the model diverge from the observations: the model simulates declines in both metrics, whereas observations indicate increases. More broadly, the model tends to produce stronger trends, both positive and negative, than the observations, resulting in greater regional variability. This is particularly evident in the model's overrepresentation of declining trends, most notable in the subpolar North, where disagreement persists at the latitudinal average scale. In contrast, most mid‐latitude and low‐latitude regions show relatively little change in both chlorophyll concentration and maximum chlorophyll concentration in both model and observations. These discrepancies suggest that PlankTOM12.2 may overestimate regional trends in concentration indices. However, these regional discrepancies are less pronounced at the global scale, where spatially averaged trends in chlorophyll concentration and maximum chlorophyll concentration are not statistically significant (Figure 3).

**FIGURE 5:**
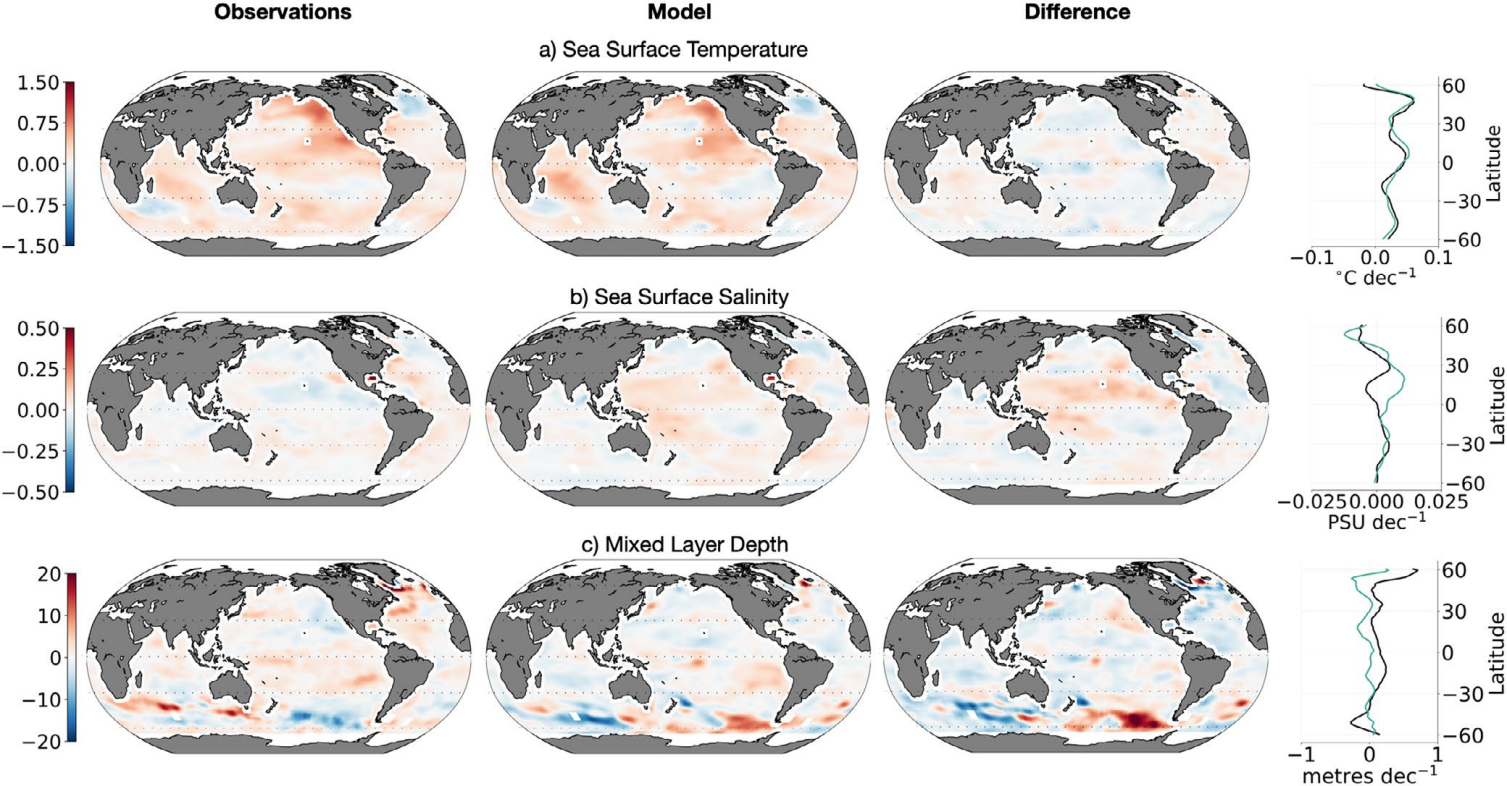
(a–c) Theil–Sen trends in physical environmental drivers calculated from 11 biennial climatologies (1999–2020). Spatial trend patterns are shown for the ARMOR3D analysis products (left), the PlankTOM12.2 model (centre), and the difference in trends (right; model—observations; red = model trend larger, blue = model trend smaller); the right‐hand panels show 10°‐smoothed zonal‐mean trends for ARMOR3D analysis products (black) and PlankTOM12.2 (green). Panels show (a) sea‐surface temperature (°C decade^−1^), (b) salinity (PSU decade^−1^) and (c) mixed‐layer depth (m decade^−1^).

These differences in phenology between the observations and PlankTOM12.2 model can originate from shortcoming in the model physical simulations, in particular those resulting from poor representation of some processes due to the coarse grid scale (e.g., eddies and frontal regions) and/or in imperfect atmospheric forcing data (e.g., winds). Regional trends in SST are consistent between the model and observations, but there is some divergence in magnitude. Modelled SST trends are larger in the Atlantic and tropical Pacific compared to the satellite‐derived observations, and smaller in the central equatorial Pacific and polar regions (Figure 5a). In contrast, important differences are noted in the representation of the modelled trends in sea surface salinity. Specifically, freshening trends observed between 0° and 15° N in the Pacific ocean are absent in the model simulation, which instead shows an increase in salinity of 0.1 PSU in this region (Figure 5b). These salinity differences propagate to differences in trends in MLD (Figure 5c). The model simulates a general stratification of the MLD over the two‐decade period, whereas observational data indicate that such shallowing is less common. Differences in salinity and MLD trends are likely contributors to the divergence in phenological responses between the model and observations. This influence is particularly evident in the tropical Pacific, where increases in maximum chlorophyll concentration coincide with regions of surface freshening and deepening MLD (Figure 4f).

### 3.4 Drivers of Phytoplankton Phenology Changes

**FIGURE 6:**
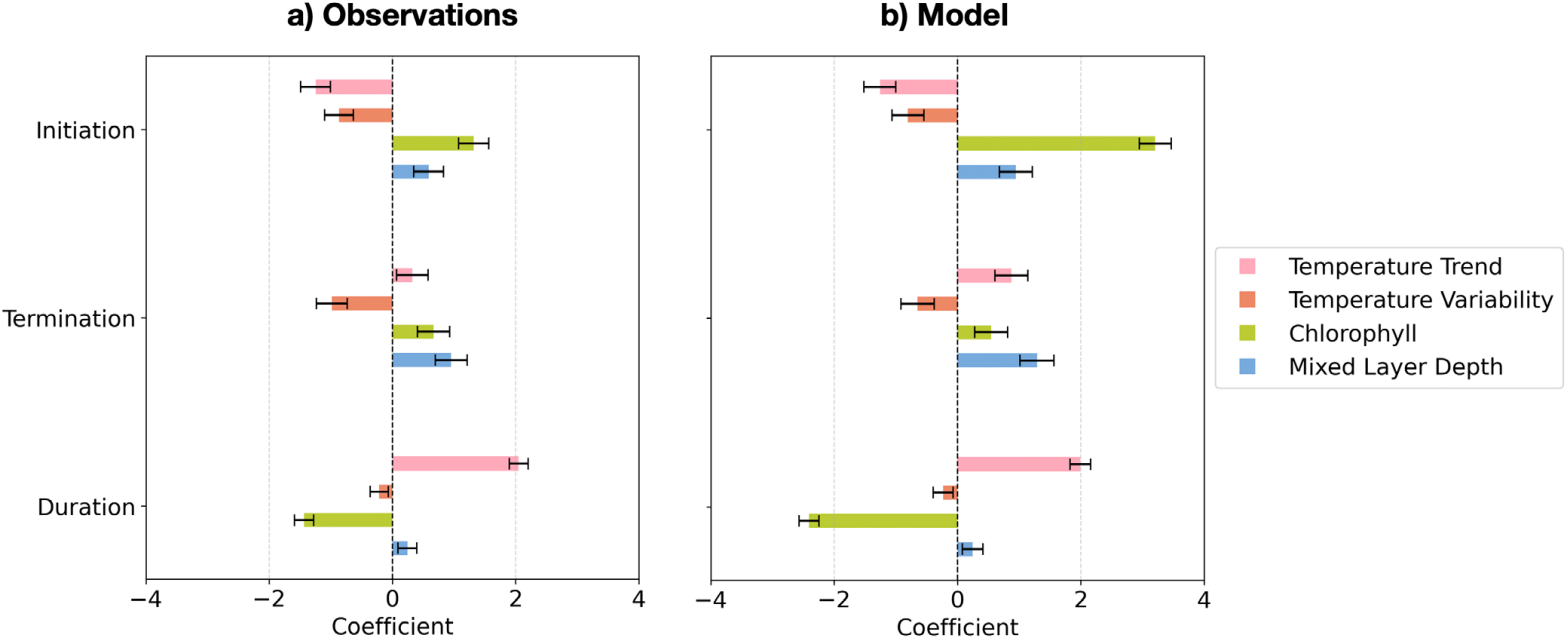
Globally averaged multiple regression coefficients for (a) observations and (b) the PlankTOM12.2 model, showing the effects of SST trend, SST variability, mean surface chlorophyll concentration, mixed‐layer depth on the phenology indices (initiation, termination and duration) between 1999 and 2020. Phenology and chlorophyll metrics in the observational analysis are derived from the OC‐CCIv5 satellite product, whereas SST and mixed‐layer depth are obtained from the Copernicus Marine Service reanalysis. Averages are calculated between 65° N and 65° S. Coefficients are expressed as the change in the phenological metric (days) per one standard deviation increase in the predictor variable given in units of days SD^−1^. All relationships are statistically significant at the *p* < 0.05 level.

In both the observations and the PlankTOM12.2 model, we calculated multiple regression relationships between phenology indices and four predictor variables: SST trends, SST variability, surface chlorophyll concentration, and MLD (Figure 6), allowing the relationship with each predictor to be evaluated while controlling for the others. Overall, the model and the observations exhibit similar magnitudes and directions of influence between these predictor variables and phytoplankton phenology.

In the satellite‐derived observations, both SST trends and SST variability are negatively associated with the initiation of the growing period, resulting in earlier initiation of $-1.25\pm 0.24\;\text{days}\;SD^{-1}$ and $-0.85\pm 0.23\;\text{days}\;SD^{-1}$, respectively, when SSTs are warmer (Figure 6). The PlankTOM12.2 model shows comparable effects, with earlier initiation of $-1.26\pm 0.25\;\text{days}\;SD^{-1}$ due to SST trends and $-0.80\pm 0.26\;\text{days}\;SD^{-1}$ due to SST variability (Figure 6). In contrast, both MLD deepening and increased chlorophyll concentrations are associated with later initiation of the growing period. In PlankTOM12.2, MLD is related to later initiation of $0.94\pm 0.26\;\text{days}\;SD^{-1}$, whereas observed MLD are related to later initiation of $0.59\pm 0.24\;\text{days}\;SD^{-1}$. Chlorophyll is related to later initiation of $3.22\pm 0.25\;\text{days}\;SD^{-1}$ in the model, compared to $1.32\pm 0.24\;\text{days}\;SD^{-1}$ in the observations. This suggests that later initiation is more strongly associated with higher chlorophyll concentrations in the model than in the observations (Figure 6).

Although SST trends and SST variability both relate to earlier initiation of the phytoplankton growing period, they have opposing influences on termination (Figure 6). SST trends are associated with a delay in termination of $0.33\pm 0.26\;\text{days}\;SD^{-1}$ and $0.88\pm 0.26\;\text{days}\;SD^{-1}$, in the observations and model, respectively. Conversely, higher temperatures due to SST variability are related to earlier termination of the growing period by $-0.97\pm 0.25\;\text{days}\;SD^{-1}$ in the observations and $-0.63\pm 0.26\;\text{days}\;SD^{-1}$ in the PlankTOM12.2 model. This suggests that whilst SST trends and variability may have contributed to the earlier initiation date observed over the last two decades, the warming SST trend appears to be the primary driver behind later termination. Increases in chlorophyll and MLD are also associated with later growing period termination in both the observations and PlankTOM12.2. Although global MLD trends is relatively small (Figure 3), regional variations can be significant (Figure 5). Our results would indicate that in regions with shallowing MLD termination would be earlier, whereas in regions with deepening MLD termination would be later (Figure 6). Furthermore, elevated chlorophyll concentration are associated with later termination in both the observations $0.66\pm 0.25\;\text{days}\;SD^{-1}$ and the PlankTOM12.2 model $0.53\pm 0.27\;\text{days}\;SD^{-1}$. Overall, SST trend, chlorophyll concentration and MLD depth show the same direction of association with termination, whereas SST variability shows the opposite pattern, advancing initiation while delaying termination.

The contrasting effects of SST trends and SST variability on termination largely determine their overall impact on the growing period's duration. A 1 SD increase in SST trend is related to an increase in the duration of the growing period by 2.04±0.15 days and 1.99±0.16 days, in the observations and model, respectively (Figure 6). In contrast, SST variability exhibits a slight negative relationship with duration, of −0.22±0.15 days and −0.23±0.16 days in the observations and model, respectively. This negative relationship arises because SST variability advances both initiation and termination to differing extents, resulting in a modest net shortening of the growing period. Moreover, chlorophyll concentration is associated with shorter growing period duration because the magnitude of the relationship with initiation is stronger than on termination. An increase of 1 SD in chlorophyll concentration is associated with later initiation by 3.22±0.25 days and later termination by only 0.53±0.27 days in the model. This results in chlorophyll concentration associated with a decrease of 2.42±0.16 days in duration (Figure 6). Finally, MLD has a small but significant positive relationship on duration in both the observations (0.25±0.15 days $SD^{-1}$) and model (0.26±0.16 days $SD^{-1}$; Figure 6).

These findings demonstrate that SST trends are the dominant predictor within the multiple regression analysis, advancing the initiation of the growing period, delaying its termination, and thereby extending its overall duration (Figure 6). In contrast, the interannual SST variability exerts a smaller, opposing impact by advancing both initiation and termination of the growing period. Chlorophyll concentration and MLD are associated with later initiation and termination of the growing period. The positive association between chlorophyll concentration and initiation timing suggests that earlier blooms tend to be associated with lower annual chlorophyll means, whereas later blooms tend to coincide with higher means. The PlankTOM12.2 model reproduces these observed relationships well, capturing the complex interactions between physical and biological drivers of phytoplankton dynamics. These findings highlight the role of long‐term SST trends in altering marine ecosystem processes and affirm the importance of incorporating both climate‐driven trends and variability into future projections of ocean phenology.

## 4 Discussion

Direct evidence of a global‐scale driver of phenological changes has been difficult to identify, despite the broad consistency across many regional studies linking phenological changes to climate change (Cooley et al. 2022). Previous studies have faced challenges in identifying a singular global driver of phytoplankton phenology, due to confounding factors and length of observational data, and challenges in detecting and attributing trends in the presence of noisy and incomplete data (Beaulieu et al. 2013; Henson et al. 2016). Furthermore, the interplay between SST, chlorophyll concentration, and MLD can mask or amplify temperature effects shaping the timing, duration, and magnitude of phytoplankton blooms at regional scales, shown here and elsewhere (Friedland et al. 2018). Here, we use a 23‐year harmonised observational dataset and apply statistical methods to separate and control for covariance among variables in order to assess their relative associations with observed phenological changes over the past two decades. Previous work has shown that climate‐related signals can emerge from approximately two decades of satellite ocean‐colour observations (Cael et al. 2023). We show that temperature trends are clearly associated with earlier initiation, later termination, and longer duration globally, whereas other variables have compounding effects. These effects are reproduced mechanistically using a global biogeochemistry model. Our results indicate that phenological trends are strongly linked to temperature warming trends at the global scale.

Although this analysis does not constitute a formal detection–attribution study, the regression framework allows the independent contributions of warming trends and interannual variability to be separated, revealing that SST trends produce a coherent lengthening of the growing period, whereas SST variability advances both initiation and termination and therefore has little net effect on duration.

Trends derived from observational phenology metrics evidenced here are consistent with previous studies (Racault et al. 2012; Zeng et al. 2013; Friedland et al. 2018; Gittings et al. 2018). Globally, the initiation of the growing period has advanced, termination has been delayed, and overall duration has increased over the past two decades. Although much of the existing literature has concentrated on changes in initiation and duration, fewer studies have highlighted trends in termination. Our results suggest that extended growing periods are associated with both earlier initiation and later termination, with both contributing in similar proportion to the trends in duration between 1998 and 2020 (Figure 3). This result is consistent with Racault et al. (2012), who, over a shorter 10‐year period (1998–2007), found that initiation dates were negatively correlated with duration, whereas termination dates were positively correlated across the global ocean. Our trends in initiation and duration are also consistent with those found by Friedland et al. (2018) at the global level over the 1998–2015 period, but they are more spatially coherent in the present study, possibly due to the longer period examined (1998–2020). Overall, despite some differences in the magnitude and nature of trends, the phenological changes identified in PlankTOM12.2 are broadly consistent with those observed in empirical datasets. These findings support the use of PlankTOM12.2‐derived phenology metrics at the global scale for examining the drivers of phenological change.

Our findings suggest that SST trends show the strongest and most consistent relationships with recent shifts in global phytoplankton phenology within the regression analysis. Between 1998 and 2020, we found increasing SSTs were related to advancing the initiation of the growing period, delaying its termination, and lengthening its duration. In contrast, the other variables examined here (SST variability, chlorophyll and MLD) all lead to shifts in initiation and termination that move in the same direction, such that both boundaries shift in parallel, hence partly cancelling their effect on duration. This pattern is reproduced in both the observational analysis and the model simulations (Figure 6). Moreover, the effect of climate variability on phytoplankton phenology has, to our knowledge, only been studied in relation to ENSO. Racault, Sathyendranath, Menon, and Platt (2017) found that during the positive phase of the Multivariate ENSO Index (MEI), the timing of phytoplankton growing periods demonstrated a diverse global impact, with both positive and negative anomalies evident across different oceanic regions. In comparison, we measure the effects of global temperature variability.

We find that increases in the temperature variability index are associated with earlier initiation, earlier termination, and slightly shorter duration globally. This pattern may be explained such that short‐term warm anomalies can advance initiation by weakening convective mixing and increasing phytoplankton residence time in the euphotic layer, consistent with mechanisms proposed for the onset of spring phytoplankton growth (Taylor and Ferrari 2011; Thomalla et al. 2015). However, such transient warming may also accelerate seasonal progression by enhancing stratification, reducing nutrient resupply, and promoting earlier nutrient limitation and/or stronger loss processes, thereby advancing termination. In contrast, sustained SST trends may shift the broader seasonal envelope, advancing the onset of favourable growth conditions while delaying the return to conditions associated with growing‐period termination. This contrast provides a mechanistic explanation for why SST variability is associated with slightly shorter growing periods, whereas sustained SST trends are associated with longer durations.

There have been previous attempts to quantify global relationships between drivers and phytoplankton phenology. Specifically, Friedland et al. (2018) investigated the effects of SST, salinity, MLD, and zonal and meridional wind stress on the initiation and duration of the primary growing period. Friedland et al. (2018) find SST trends were not correlated with bloom initiation and negatively correlated with bloom duration over 5° latitudinal bins. In contrast, we find that temperature trends are related to advances in initiation, delays in termination and subsequentially lengthening of the duration of the growing period. The disparity between our findings and those reported by Friedland et al. (2018) could stem from several methodological differences. We use a significantly longer dataset of satellite chlorophyll concentration data (23 years, compared to 17 years). Our methodology accounts for confounding or opposing variables, whereas that of Friedland et al. (2018) does not, and hence the effect of SST trends could have been masked by the presence of variability in the other drivers, especially given the shorter time period. As evidenced in Figure 6, the effects of chlorophyll, MLD, and SST variability often counteract the effect of trends in SST. The extent to which methodological differences in the computation of the phenological indices (see Section 2) might also contribute to the contrasting results is uncertain. The use of the OC‐CCIv5 harmonised chlorophyll dataset could also have improved the spatial coherence in the results found here.

Previous studies have also identified MLD to be a crucial driver in phytoplankton phenology. Warming can lead to earlier and stronger stratification, reducing nutrient replenishment but simultaneously increasing light availability in the surface layer, thereby promoting earlier bloom initiation at high latitudes (Silva et al. 2021). High‐latitude growing periods are influenced by light limitation and predator dilution, whereas termination often stems from increased grazing and nutrient limitation (Racault et al. 2012; Behrenfeld et al. 2013; Sallée et al. 2015; Ardyna and Arrigo 2020; Thomalla et al. 2023). In tropical regions, shallower MLD decreases nutrient availability, shortening growing periods due to nutrient depletion, whereas deeper MLD can delay the termination of growing periods, extending their duration (Gittings et al. 2018; Racault et al. 2012; Krug et al. 2018; Friedland et al. 2018). Our global results support these findings, demonstrating that the global deepening MLD between 1999 and 2020 are related to later initiation and later termination dates, and consequently in only marginally longer duration of the growing period due to the compensating effects, although we do not specifically attribute the relative contributions of light, nutrient availability, or grazing within this analysis (Figure 6).

## 5 Conclusion

This study identifies SST trends as the strongest predictor of recent global shifts in marine phytoplankton phenology among the variables examined, supported by both statistical analysis and mechanistic model experiments. Observational evidence from chlorophyll‐based satellite data reveals consistent trends towards earlier bloom initiation, later termination, and longer growing periods across diverse oceanic regions. The short duration of chlorophyll records has historically limited our ability to attribute these changes to specific drivers. The availability of the OC‐CCIv5 harmonised chla product covering over than two decades has enabled a longer, more coherent analysis. The analysis is reinforced by the use of the PlankTOM12.2 global ecosystem model, which captures complex biological and physical processes, and its successful replication of the observed phenological trends and global relationships with underlying drivers. The strong agreement between model outputs and satellite‐derived observations, combined with established mechanistic links between temperature and phytoplankton phenology and the absence of trends in the constant‐climate simulation, strengthens confidence in SST trends as the dominant driver of the observed phenological shifts.

By distinguishing the effects of long‐term SST trends from short‐term variability and localised biophysical conditions, this model‐data synthesis resolves previous uncertainties regarding the drivers of phenological change. Previous work highlighted diverse local factors ranging from nutrient availability to grazing intensity, yet did not isolate a consistent controlling mechanism across different ocean basins. Here, our model‐data comparison shows that ongoing warming may alter bloom timing and intensity beyond the compensatory effects of SST variability, MLD or chlorophyll. Our findings indicate that global ocean warming emerges as the strongest predictor of phytoplankton phenological change relative to SST variability, mixed‐layer depth, and chlorophyll concentration.

Overall, our findings support the current consensus from local studies showing that 78% of long‐term (> 19 years) phytoplankton phenology studies report shifts consistent with climate change (Cooley et al. 2022), and demonstrate that these local findings reflect global patterns in the response of phenological indices to ocean warming.

## Author Contributions

**Corinne Le Quéré:** funding acquisition, writing – review and editing, supervision, conceptualization, methodology, resources. **Joe K. Guest:** conceptualization, investigation, methodology, validation, visualization, writing – original draft, formal analysis, writing – review and editing. **Marie‐Fanny Racault:** supervision, writing – review and editing, conceptualization, methodology.

## Conflicts of Interest

The authors declare no conflicts of interest.

## Data Availability

All data required to reproduce the analysis as well as the phenology metrics produced by the data are archived at Zenodo: https://zenodo.org/records/17368092. This dataset contains all environmental and biological fields used in the study, including processed output from the PlankTOM12.2 global marine biogeochemical model and observationally derived products, provided on a consistent 1° × 1° global grid for the period 1998–2020. Model output includes chlorophyll‐a concentration (TCHL), sea surface temperature (tos), sea surface salinity (sos), and mixed‐layer depth (somxl030). Full model description and equations are provided by Buitenhuis et al. (2023), and further details on the model configuration are available in Guest (2023). Physical driver fields are derived from the ARMOR3D global ocean reanalysis product, which combines satellite and in situ observations through statistical interpolation. The construction of these fields is described by Guinehut et al. (2012) and Mulet et al. (2012). Preprocessing steps applied in this study are documented within the Zenodo archive. Satellite chlorophyll‐a data are based on the ESA Ocean Colour Climate Change Initiative (OC‐CCI) v5.0 product. Due to data distribution constraints, the raw OC‐CCI data must be downloaded separately from the official repository. A fully reproducible processing workflow, including bias correction, spatial aggregation, gap filling, and temporal smoothing, is provided within the Zenodo archive and at the project repository: https://github.com/joe‐kguest/global‐phytoplankton‐phenology. All derived datasets and processing steps necessary to reproduce the results and figures are included in the Zenodo archive and github repository.
